# An Efficient Conformal Stacked Antenna Array Design and 3D-Beamforming for UAV and Space Vehicle Communications

**DOI:** 10.3390/s21041362

**Published:** 2021-02-15

**Authors:** Yasser Albagory

**Affiliations:** 1 Department of Computer Engineering, College of Computers and Information Technology, Taif University, P.O. Box 11099, Taif 21944, Saudi Arabia; y.albagory@tu.edu.sa; 2 Electronics and Electrical Communications Engineering Department, Faculty of Electronic Engineering, Menoufia University, Menouf 32952, Egypt

**Keywords:** antenna arrays, unmanned aerial vehicles, adaptive arrays, space vehicles

## Abstract

In this paper, a new conformal array structure and beamforming technique are proposed to provide efficient communication performance for unmanned aerial vehicles (UAVs) and space vehicles. The proposed array is formed by conformally stacking cylindrical, conical, and concentric circular (CSC^4^) arrays which are all coaxially aligned with the same axis of the conformed body and with uniform interelement spacing. The array elements are then fed by a weighting vector that has an adaptive cosine tapered profile where the maximum amplitude coefficient is oriented with the mainlobe direction to improve the scanning capabilities of the array and increase the array effective area. In addition, for very large, conformed body structures such as space vehicles, a frontal mainlobe-oriented partial CSC^4^ array beamforming technique is proposed to efficiently utilize the large CSC^4^ structure, reduce the processing requirements for mainlobe electronic steering, and to provide very low sidelobe levels with reduced backlobe levels. Simulation results show that the proposed CSC^4^ design can provide wide scanning angles of up to ±70° angular range in the θ-direction with only ±1° change in the beamwidth, without increasing array size and with achievable sidelobe level of −45 dB and backlobe levels less than −10 dB.

## 1. Introduction

### 1.1. Background and Motivation

Antenna arrays are very essential for many communication systems, including wireless networks, mobile, satellite, radar, remote sensing, and many other applications [1,2,3]. Recently, antenna arrays have had a main role in pushing the data rates and system capacity to very high levels using efficient beamforming techniques. The array structure has a very important role in optimizing the system performance and determines the beamforming capabilities. Several array structures can be applied in various applications including linear, two-dimensional, circular, conical, cylindrical, spherical, and even nonuniform array structures [3]. However, some applications require specific designs in which the antenna array should be fixed on the body structure, such as airborne systems, missiles, unmanned aerial vehicles (UAVs), space shuttles, rockets, and even maybe on the surface of satellite fairings [4,5,6,7]. Most of these structures have a tapered front shape which is almost configured as cylindrical with conical tips to improve the aerodynamic performance and reduce wind resistance. Additionally, UAVs and space vehicles are always subjected to continuous movements with changing direction and rotation, so, fixing antenna arrays on their body should be manipulated carefully to maintain continuous communication link availability during flight. In addition, the very long distance spanned by space vehicles, such as those travelling to Mars, requires high gain and efficient adaptive conformal antennas to ensure high quality of the communication link. Several conformal antenna array structures have been proposed in the literature [4,5,6,7,8,9,10,11,12,13,14,15,16,17,18,19,20,21,22,23,24,25,26,27,28,29,30,31], where most of them are fabricated in the form of microstrip patch antennas printed on a conical surface [8,9,10,11,12,13,14] or slotted cylindrical and conical structures [15,16]. For example, in [17], a conical array was designed for radar applications using genetic algorithm to reduce sidelobe levels. In [18], a switched beam conformal conical array was proposed, using 12 elements at 5.2 GHz. For UAV communications, the work done in [19] proposed conical conformal array at 9.8 GHz for point-to-point communications, while in [20], the cylindrical antenna arrays are combined with conical arrays to form “CYLCON” array using microstrip Yagi antennas to provide wide solid angle scanning. On the other hand, the recent work done in [30] discussed the radiation properties of different source geometries based on the inverse source problem. One of the most important achievements in [30] is the focused patterns for mainlobes at different angular directions, however, the tested structures have relatively high sidelobe levels. Additionally, spherical conformal arrays were proposed in [31] to compensate for the changing spherical surfaces using patch antenna structures. However, these structures lack the flexible adaptive beamforming capabilities for the radiation pattern optimization. For example, it is desired to reduce the secondary backlobe radiation of the array, reduce sidelobe levels, maintain a constant beamwidth over wide scanning angles, and provide fast weight calculation and processing. These requirements are sought after for fast-moving bodies such as UAVs and space vehicles. In addition, there should be an efficient utilization of the elements according to the mainlobe directions, especially at the endfire direction where the projection of the array is the minimum. In addition, a UAV requires powerful signals for long-range communication, control, and telemetry, while the consumed energy should be minimized. For space vehicles, and during flight, the communication link should be powered by high-gain antenna arrays before deploying the spot-beam antennas of the satellite inside fairing prior to the releasing operation to improve the control and telemetry performance, especially at very large communication distances. The communication link of these two applications requires flexible adaptive electronic steering to avoid link loss due to body rotation. 

### 1.2. Paper Contributions

To achieve the goals mentioned in the previous section, this paper proposes a conformal stacked structure of three specific array configurations which are cylindrical, conical, and concentric circular (CSC^4^) arrays that fit UAV and space vehicle geometrical body structures. The array structure is geometrically analyzed to deduce the general composite array steering vector, which is essential for beamforming operation. The array performance is examined for uniform and adaptive feeding in which a weighting function is proposed for efficient sidelobe and backlobe reduction. On the other hand, the proposed adaptive weights in this paper are designed with mainlobe vector orientation property where the maximum weights are always maintained at the antenna elements lying on or nearest to the mainlobe direction on the array surface. This property provides the smallest beamwidth variation over wide scanning angles and reduces the projection effects on the radiation pattern near to the endfire direction. In addition, the self-body blocking problem of the conformal array fixed on a large body structure is manipulated by proposing a frontal mainlobe-oriented partial subarray beamforming technique, in which a partial array is extracted from the whole CSC^4^ array through steering vector and is examined to provide very deep sidelobe levels with reduced backlobe levels.

### 1.3. Paper Contributions

After Section 1 and Section 2 models the individual conformal array structures to explore the impact of each configuration on the system performance. Section 3 proposes the conformal stacked cylindrical conical and concentric circular (CSC^4^) array, while Section 4 investigates the frontal mainlobe-oriented partial CSC^4^ array. In Section 5, the practical challenges of the proposed array are explored and discussed, and, finally, Section 6 concludes the paper and provides the related future work.

## 2. Modeling of Individual Array Structures

In this section, the uniform conical, cylindrical, and concentric circular arrays are modeled individually, where each array structure is analyzed geometrically using isotropic antenna elements to generally explore the impact of the proposed beamforming technique. The uniform conical array is first designed, which forms most of the stacked array structure. Then, this conical array can be reconfigured simply to obtain the design of cylindrical and concentric circular structures. Additionally, the radiation properties of each array are demonstrated individually before forming the complete CSC^4^ to explore the impact of each array on the overall radiation pattern.

Other array structures, such as two-dimensional array, are not considered in this paper because they do not possess the conformal properties required for UAV and space vehicle structures and the azimuthal symmetry required in the 3D beamforming.

### 2.1. Uniform Conical Array

**Figure 1:**
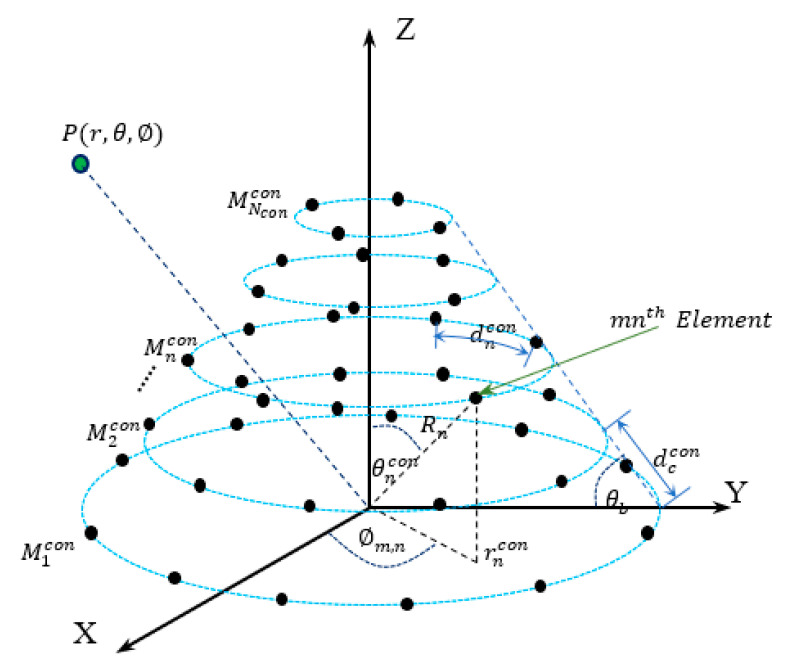
Conical array structure.

In this subsection, the uniform conical antenna array is modeled. Assuming that a conical array of circular base that lies in the XY-plane with its center at the origin, the antenna elements are assumed to be isotropic radiators with neglected mutual coupling effects. The conical array is formed by adding or stacking circular ring arrays that should be coaxially centered around the *Z*-axis, as shown in Figure 1, with uniform separation distance between the ring contours. These coaxial rings shrink in diameter according to the base angle of the conical array, $\theta_{b}$, where the *n^th^* ring contour is directly separated from its neighbored ones by a slant distance $d_{c}^{con}$, has a radius $r_{n}^{con}$ measured horizontally from the *Z*-axis, and has a number of elements given by $M_{n}^{con}$. 

The neighboring elements in the *n^th^* ring are also separated approximately by a distance $d_{n}^{con}$ which is given by:

(1) $$d_{n}^{con}=\frac{2\pi r_{n}^{con}}{M_{n}^{con}}$$

As shown in Figure 1, each upper ring array has a radius that is less than its lower one by a distance $d_{c}^{con}cos\left(\theta_{b}\right)$, and the *n^th^* ring radius $r_{n}^{con}$ is given by:

(2) $$r_{n}^{con}=r_{1}^{con}-\left(n-1\right)d_{c}^{con}cos\left(\theta_{b}\right)$$

where $r_{1}^{con}$ is the radius of the base ring of the conical array. The number of elements of the *n^th^* ring can be also determined from the number of elements of the base ring array $M_{1}^{con}$, as follows:

(3) $$M_{n}^{con}=\frac{2\pi }{d_{n}^{con}}\left(\frac{M_{1}^{con}d_{1}^{con}}{2\pi }-\left(n-1\right)d_{c}^{con}\cos\left(\theta_{b}\right)\right)$$

or

(4) $$M_{n}^{con}=\frac{M_{1}^{con}d_{1}^{con}}{d_{n}^{con}}-\frac{2\pi \left(n-1\right)d_{c}^{con}\cos\left(\theta_{b}\right)}{d_{n}^{con}}$$

For uniform conical array, we assume that $d_{n}^{con}=d_{c}^{con}$ for all rings where all elements in the array are separated by the same distance. Therefore, the number of elements in the nth ring becomes:

(5) $$M_{n}^{con}=M_{1}^{con}-2\pi \left(n-1\right)\cos\left(\theta_{b}\right)$$

As the number of antenna elements in any of the array rings must be an integer number, the second term in Equation (5) should be also an integer number to achieve the uniformity of the conical array or:

(6) $$2\pi \cos\left(\theta_{b}\right)=l$$

where l is an integer number that must satisfy the following inequality:

(7) l<2π

Therefore, the possible set of values of l and the corresponding base angles are shown in Table 1. However, any other value for the base angle can be used, which results in minor perturbations in the conical surface of the array due to the approximation done in Equation (5) or slight variation in the interspacing distances. On the other hand, the nth ring in the conical array has a conical contour angle $\theta_{n}$ which is given by:

(8) $$\theta_{n}^{con}=\tan^{-1}\left(\frac{r_{n}^{con}}{\left(n-1\right)d_{c}^{con}\sin\left(\theta_{b}\right)}\right)$$

This angle has an important role in finding the array steering vector, which is essential in the beamforming operation. 

### 2.2. Design of Uniform Cylindrical and Concentric Circular Arrays Using Uniform Conical Array Parameters

The design of uniform conical array in the previous subsection can be useful also in designing the uniform cylindrical and concentric circular arrays by setting the value of $\theta_{b}$ to the two boundary values, which are 90° and 0°. Therefore, if $\theta_{b}=90{}^{\circ}$, with a limited number of rings, the uniform cylindrical array will be obtained where its parameters are given as follows:

(9) $$M_{n}^{cyl}=M_{1}^{cyl},$$

(10) $$r_{n}^{cyl}=r_{1}^{cyl},$$

and

(11) $$\theta_{n}^{cyl}=\tan^{-1}\left(\frac{r_{1}^{cyl}}{\left(n-1\right)d_{c}^{cyl}}\right)$$

where $r_{1}^{cyl}=\frac{M_{1}^{cyl}d_{1}^{cyl}}{2\pi }$.

The array parameters for uniform concentric circular array can be obtained also by setting $\theta_{b}=0{}^{\circ}$ as follows:

(12) $$M_{n}^{cc}=M_{1}^{cc}-2\pi \left(n-1\right)$$

(13) $$r_{n}^{cc}=r_{1}^{cc}-\left(n-1\right)d_{c}^{cc}$$

and

(14) $$\theta_{n}^{cc}=90{}^{\circ}$$

where $r_{1}^{cc}=\frac{M_{1}^{cc}d_{1}^{cc}}{2\pi }$. 

For concentric circular array design, Equation (12) is subjected to an approximation when finding the number of elements of the inner ring arrays. The approximation is applied to “2π” which should be an integer number, so it can be approximated to 6, and the array elements in the inner rings are therefore decreasing gradually by 6 elements, and correspondingly Equation (12) can be rewritten as follows:

(15) $$M_{n}^{cc}=M_{1}^{cc}-6\left(n-1\right)$$

The angular distance separation between elements in the rings resulted from Equation (15) is slightly higher than the inter-ring separation distance as follows:

(16) $$d_{c}^{cc}\approx 0.995\;d_{n}^{cc}$$

Equation (15) is useful for an inward concentric circular array design which starts from the outermost ring and determines the sizes of the inner rings. However, for centrally outward design, we may use the following formula:

(17) $${M^{\prime }}_{n}^{cc}={M^{\prime }}_{1}^{cc}+6\left(n-1\right)$$

where ${M^{\prime }}_{1}^{cc}$ is the number of elements in the innermost ring of the uniform concentric circular array, which can be set to 1 if we want to design a uniform concentric ring array with central element feeding. The total number of elements in each of the three array designs can be calculated as follows:

(18) $$\eta_{T}^{con}=N^{con}M_{1}^{con}+\overset{N^{con}}{\underset{n=2}{\sum }}l\left(1-n\right)$$

(19) $$\eta_{T}^{cyl}=N^{cyl}M_{1}^{cyl}$$

(20) $$\eta_{T}^{cc}=N^{cc}{M^{\prime }}_{1}^{cc}+\overset{N^{cc}}{\underset{n=2}{\sum }}6\left(n-1\right)$$

where $N^{con}$, $N^{cyl}$, and $N^{cc}$ are the numbers of rings in the conical, cylindrical, and concentric circular arrays, respectively.

### 2.3. General Array Steering Vector

In this section, a general array steering vector is deduced that can be applied to any of the three individual array structures. As shown in Figure 1, a plane wave originating from a source at point P impinges on the array elements with different phases according to the distance between P and the antenna and the relative position of antenna elements with the origin point. The array steering vector contains all phase responses of the elements, which is essential in determining the spatial power response of the array for an element that is located on the array surface at a spherical coordinate $\left(\theta_{n},\emptyset_{m,n},R_{n}\right)$ where $\theta_{n}$ is measured between the *Z*-axis and the radial distance $R_{n}$ to the element located on the nth ring, and $\emptyset_{m,n}$ is the azimuth angle of this element. As the three arrays are assumed to be symmetric around the *Z*-axis, the radial distance to any element located on the same ring is the same, and is given by:

(21) $$R_{n}=\frac{r_{n}}{\sin\left(\theta_{n}\right)}$$

The phase response of the mnth element to the impinging signal relative to the origin point can be deduced directly from Figure 1 and is given by [32]:

(22) $$a_{m,n}\left(\theta ,\phi \right)=e^{j\frac{2\pi }{\lambda }R_{n}\psi_{m,n}}$$

where:

λ is the signal wavelength,
$\psi_{m,n}=\zeta_{1}+\zeta_{2}+\zeta_{3}$,
$\zeta_{1}=\sin\left(\theta \right)\cos\left(\phi \right)\sin\left(\theta_{n}\right)\cos\left(\phi_{m,n}\right)$,
$\zeta_{2}=\sin\left(\theta \right)\sin\left(\phi \right)\sin\left(\theta_{n}\right)\sin\left(\phi_{m,n}\right)$,
$\zeta_{3}=\cos\left(\theta \right)\cos\left(\theta_{n}\right)$,

and the overall steering vector can be built by concatenating the steering responses for each ring, starting from the bottom ring which lies in the XY-plane, as follows:

(23) $$A\left(\theta ,\phi \right)={\left[a_{1}\left(\theta ,\phi \right),a_{2}\left(\theta ,\phi \right),\ldots ,a_{N}\left(\theta ,\phi \right)\right]}^{T}$$

where

(24) $$a_{n}\left(\theta ,\phi \right)=\left[e^{j\frac{2\pi }{\lambda }R_{n}\psi_{1,n}}\;e^{j\frac{2\pi }{\lambda }R_{n}\psi_{2,n}}\;\ldots \;e^{j\frac{2\pi }{\lambda }R_{n}\psi_{M_{n},n}}\right]$$

is the nth ring steering vector and $M_{n}$ is the corresponding number of elements. 

Assuming that W is the array weighting vector, then the array power pattern, $G_{p}\left(\theta ,\phi \right)$, can be written as follows:

(25) $$G_{p}\left(\theta ,\phi \right)={\left|W^{H}A\left(\theta ,\phi \right)\right|}^{2}$$

where *H* is the Hermitian operator. 

To compare between the properties of the 3D radiation patterns of the three array structures, we may apply uniform feeding where all the amplitude coefficients in W are equal to 1 with the same phase response as the array steering vector at the desired direction according to (25) or:

(26) $$W_{uni}=A\left(\theta_{o},\phi_{o}\right)$$

The design parameters of each array are shown in Table 2, where the design parameters are adjusted so that the total number of elements of each array is constant and the interelements separation is λ/2. 

**Figure 2:**
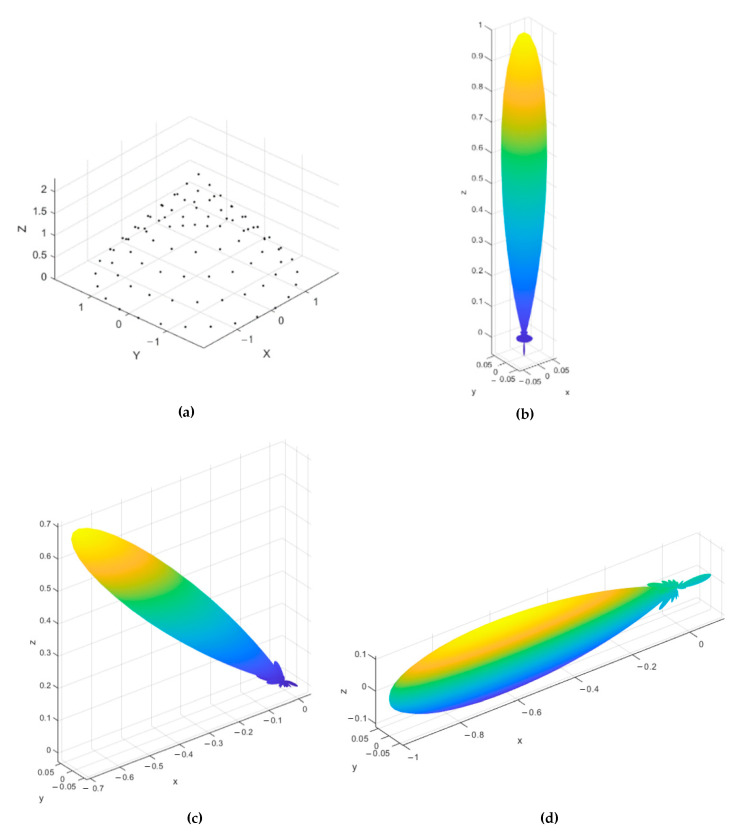
Uniform conical array structure and beam characteristics at three mainlobe directions: (**a**) array structure, (**b**) normalized 3D power pattern for a mainlobe at (0°,180°), (**c**) normalized 3D power pattern for a mainlobe at (45°,180°), and (**d**) normalized 3D power pattern for a mainlobe at (90°,180°).

**Figure 3:**
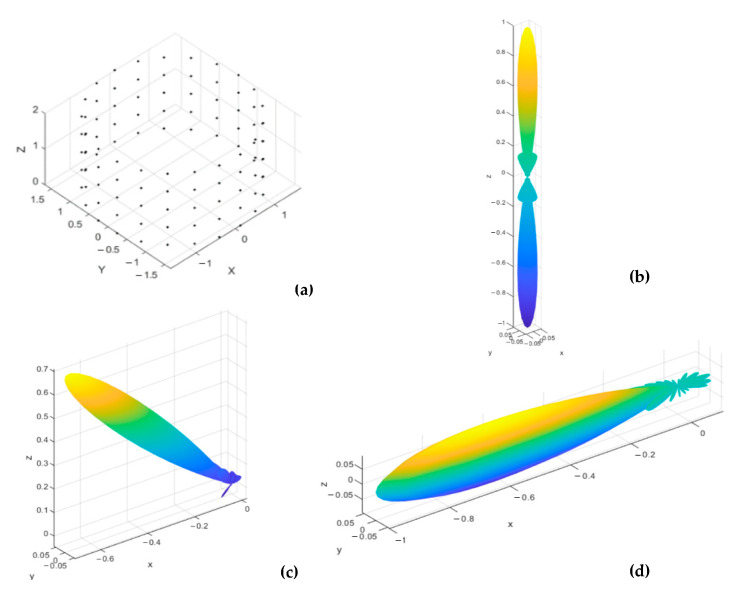
Uniform cylindrical array structure and beam characteristics at three mainlobe directions: (**a**) array structure, (**b**) normalized 3D power pattern for a mainlobe at (0°,180°), (**c**) normalized 3D power pattern for a mainlobe at (45°,180°), and (**d**) normalized 3D power pattern for a mainlobe at (90°,180°).

**Figure 4:**
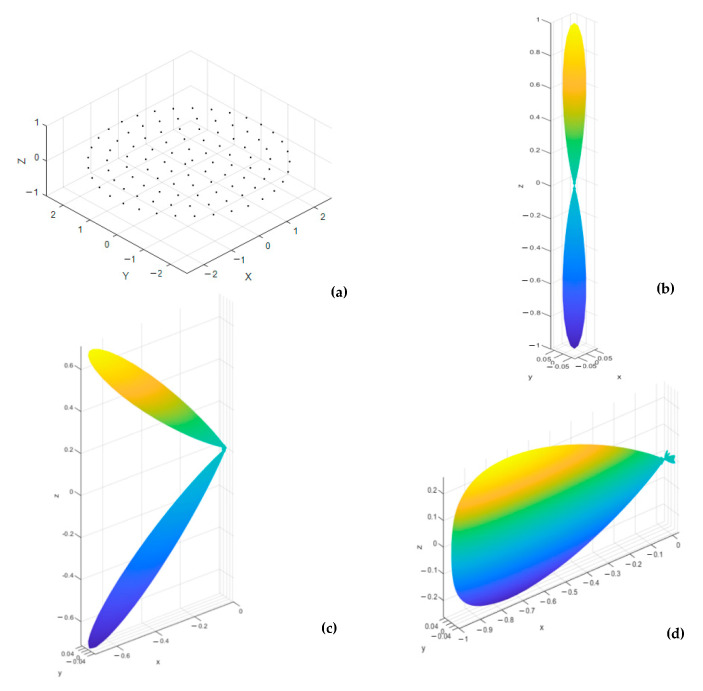
Uniform concentric circular array structure and beam characteristics at three mainlobe directions: (**a**) array structure, (**b**) normalized 3D power pattern for a mainlobe at (0°,180°), (**c**) normalized 3D power pattern for a mainlobe at (45°,180°), and (**d**) normalized 3D power pattern for a mainlobe at (90°,180°).

The three configurations, along with their corresponding normalized power pattern, are shown in Figure 2, Figure 3 and Figure 4, where three mainlobe directions, (0°,180°), (45°,180°), and (90°,180°), are used for testing the 3D radiation properties of each array. The conical array provides the lowest backlobe level at any mainlobe direction, as shown from Figure 2, while cylindrical and concentric circular arrays possess symmetric backlobe with the mainlobe, especially at the broadside mainlobe direction. The backlobe level at higher values of $\theta_{o}$ will be reduced in the cylindrical array, especially near $\theta_{o}=90{}^{\circ}$, and appears again when $\theta_{o}=180{}^{\circ}$. On the other hand, the concentric circular array provides an image backlobe of the same level as of the mainlobe at any value of $\theta_{o}$. However, the concentric circular array provides the narrowest beamwidth compared to the other arrays, except near $\theta_{o}=90{}^{\circ}$ where the mainlobe and its image combine into a wider beam. The cylindrical array provides smaller beamwidth than the conical array but suffers from the higher backlobe and sidelobe levels. The beamwidth of cylindrical array is greater than that of the conical array for most θ≤45° directions, after which the broad face area of the cylindrical array provides the smallest beamwidth, among other designs. From the 3D normalized power patterns of the three arrays, a very attractive feature in the conical array is the reduced levels of the sidelobes and backlobe, which is very important in many applications that are sensitive to the interference. 

We can conclude from this simple analysis of the three individual structures that when we design a composite conformal array structure, we can predict the effect of increasing the size of each array on the overall power pattern performance, as is demonstrated in the next section. The individual element directivity pattern also has a very important role in choosing the proper composite structure. 

## 3. Conformal Stacked Cylindrical Conical Concentric Circular (CSC^4^) Array Structure and Beamforming

### 3.1. Structure and Steering Vector of CSC^4^

**Figure 5:**
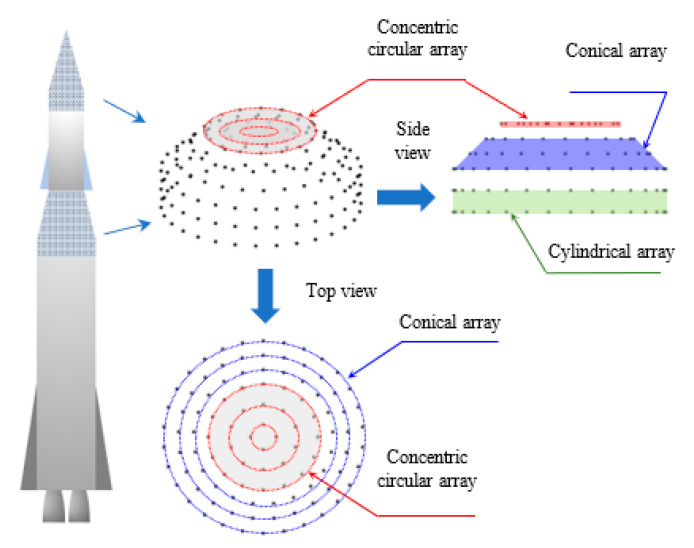
Concentric circular (CSC^4^) antenna array structure.

The body structure and geometry of most airborne vehicles, space shuttles, rockets, missiles, and airplanes possess tapered tips that are especially designed in such a way that takes into consideration the aerodynamics and air resistance. Therefore, conical and cylindrical antenna arrays are suitable and conform to these body structures. In addition, the array structures shown in the previous section can be combined into a stacked modular one to gain most of their advantages while conforming to these flying body structures. The proposed combined or stacked structure can be formed in a modular add-on fashion, as shown in Figure 5, where the cylindrical array forms the base of the structure followed by conical array, then the concentric circular array completes the design at the tip end where it can be fixed in the radome nose of the aircraft or space vehicle. This modular structure is expected to minimize the levels of unwanted sidelobes in the radiation pattern, improve beamwidth performance with direction, provide wider scanning angles than any one of the individual conventional arrays, and provide beams with high front-to-back ratios. In [20], a similar geometrical structure is formed by only conical and cylindrical arrays with nonuniform array interspacing of microstrip Yagi antenna arrays. However, in this paper, the concentric circular array is added at the top of the array to improve the frontal directivity with the adaptive beamforming capabilities for steering the mainlobe over a wider scanning angle and with uniform antenna elements distribution.

Assuming that the array elements are in the form of isotropic radiators, the overall CSC^4^ array steering vector can be segmented into three subvectors, as follows:

(27) $$A_{S}\left(\theta ,\phi \right)=\left[\begin{array}{c} A_{cyl}\left(\theta ,\phi \right)\\ A_{con}\left(\theta ,\phi \right)\\ A_{cc}\left(\theta ,\phi \right) \end{array}\right]$$

where each subvector in $A_{S}\left(\theta ,\phi \right)$ corresponds to one of the array structures and depends on the corresponding antenna elements locations, as follows:

(28) $$A_{cyl}\left(\theta ,\phi \right)=[\begin{array}{c} a_{1}^{cyl}\left(\theta ,\phi \right)\\ a_{2}^{cyl}\left(\theta ,\phi \right)\\ :\\ a_{N_{cyl}}^{cyl}\left(\theta ,\phi \right) \end{array}]$$

(29) $$a_{n}^{cyl}\left(\theta ,\phi \right)={\left[e^{j\frac{2\pi }{\lambda }R_{n}\psi_{1,n}}\;e^{j\frac{2\pi }{\lambda }R_{n}\psi_{2,n}}\ldots \;e^{j\frac{2\pi }{\lambda }R_{n}\psi_{M_{n}^{cyl},n}}\right]}^{T},\;n=1,2,\;\ldots ,\;N_{cyl}$$

(30) $$A_{con}\left(\theta ,\phi \right)=\left[\begin{array}{c} a_{N_{cyl}+1}^{con}\left(\theta ,\phi \right)\\ a_{N_{cyl}+2}^{con}\left(\theta ,\phi \right)\\ :\\ a_{N_{cyl}+N_{con}}^{con}\left(\theta ,\phi \right) \end{array}\right]$$

$a_{n}^{con}\left(\theta ,\phi \right)={\left[e^{j\frac{2\pi }{\lambda }R_{n}\psi_{1,n}}\;e^{j\frac{2\pi }{\lambda }R_{n}\psi_{2,n}}\ldots \;e^{j\frac{2\pi }{\lambda }R_{n}\psi_{M_{n}^{con},n}}\right]}^{T}$,

(31) $$n=N_{cyl}+1,N_{cyl}+2,\;\ldots ,\;N_{cyl}+N_{con}$$

(32) $$A_{cc}\left(\theta ,\phi \right)=\left[\begin{array}{c} a_{N_{cyl}+N_{con}+1}^{cc}\left(\theta ,\phi \right)\\ a_{N_{cyl}+N_{con}+2}^{cc}\left(\theta ,\phi \right)\\ :\\ a_{N_{cyl}+N_{con}+N_{cc}}^{cc}\left(\theta ,\phi \right) \end{array}\right]$$

$a_{n}^{cc}\left(\theta ,\phi \right)={\left[e^{j\frac{2\pi }{\lambda }R_{n}\psi_{1,n}}\;e^{j\frac{2\pi }{\lambda }R_{n}\psi_{2,n}}\ldots \;e^{j\frac{2\pi }{\lambda }R_{n}\psi_{M_{n}^{cc},n}}\right]}^{T}$,

(33) $$n=N_{cyl}+N_{con}+1,\;\ldots ,\;N_{cyl}+N_{con}+N_{cc}$$

The choice of $N_{cyl}$, $N_{con}$, and $N_{cc}$ depends on several factors, such as the geometry and dimensions of the body over which the array is fixed, the scanning range of the mainlobe, the mainlobe absolute gain and the relative sidelobe and backlobe levels, and the mainlobe direction, especially the elevation angle $\theta_{o}$. The azimuth angle $\phi_{o}$ does not affect greatly the power pattern due to the symmetry of the array around the *Z*-axis. It is recommended to reduce the size of the concentric circular array to reduce the backlobe level while increasing the conical array size to reduce the backlobe radiation. On the other hand, the cylindrical array size should be increased for beams directed towards the endfire direction of the array to reduce the beamwidth. However, for broadside directed mainlobes, the cylindrical array size should be decreased to reduce the backlobe radiation level. Therefore, the overall array size can be adapted according to the mainlobe direction to optimize the radiation pattern by controlling the cylindrical array size so that it can be reduced for frontal broadside mainlobe directions, while increased for those mainlobe directions near to the endfire. 

### 3.2. Uniformly Fed CSC^4^ Array Performance

**Figure 6:**
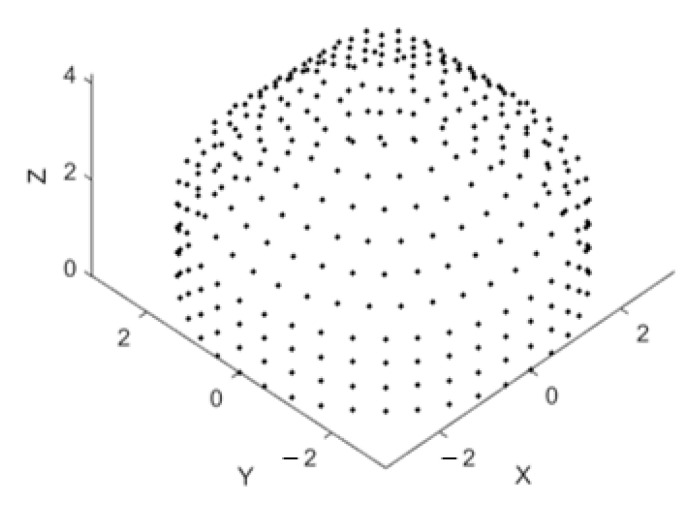
Arbitrary design of uniform CSC^4^ antenna array using the parameters shown in Table 3. The axes are normalized to the wavelength.

Firstly, the proposed array structure is tested using uniform feeding, then the next section smooths the pattern by applying an adaptive tapered feeding profile to reduce the sidelobe levels. As shown in Figure 5, a CSC^4^ array is designed so that it can be assembled in layered structure, and the only part that can be adapted in the array is the number of rings of the cylindrical array and the feeding currents. A conical array can be used completely without any topping of concentric circular arrays, or may be trimmed at its top to insert the concentric circular array for beamwidth reduction. With the design parameters listed in Table 3, a uniform CSC^4^ is shown in Figure 6, where the coordinate axes are normalized to λ. 

**Figure 7:**
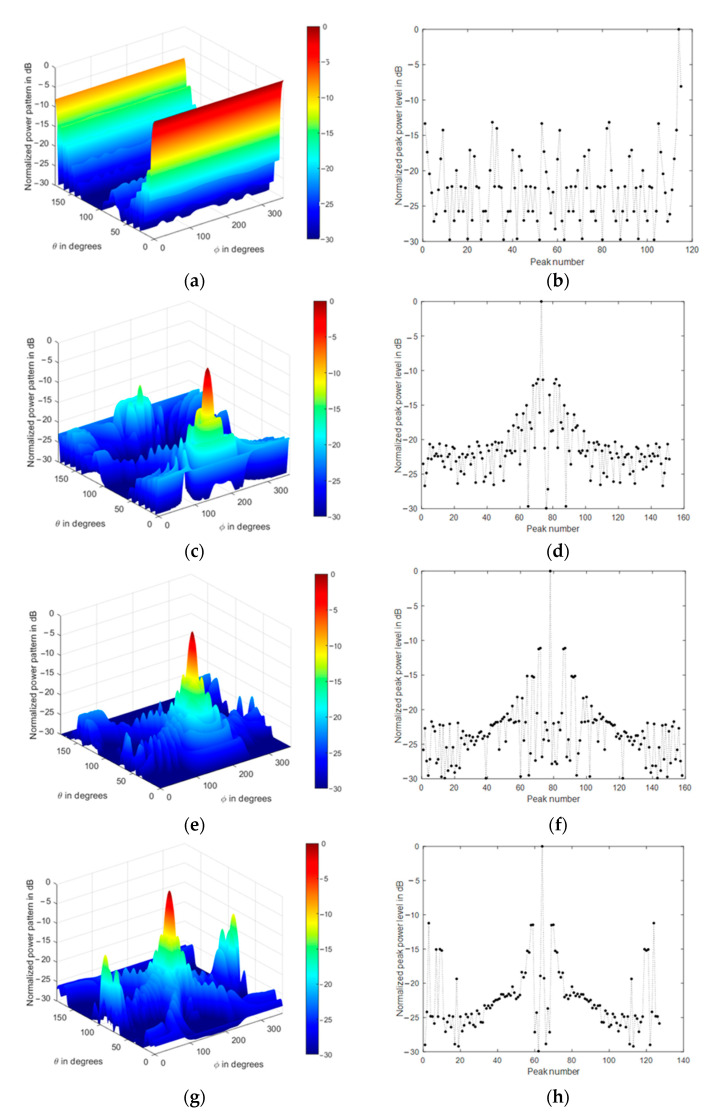
Normalized power pattern of uniformly fed CSC^4^ (**a**,**c**,**e**,**g**) and the corresponding radiation peaks (**b**,**d**,**f**,**h**) at four different mainlobe directions (0°,180°), (30°,180°), (60°,180°), (90°,180°), respectively.

The array normalized power pattern is demonstrated at four different mainlobe directions, as shown in Figure 7a–d, where the impact of the conical array on the backlobe levels reduction is clear. Additionally, the corresponding radiation peaks are shown in Figure 7e–h, where they include the mainlobe (0 dB), sidelobes, and backlobe peaks.

The radiation peaks are simply obtained by finding the local maximum values of the radiation lobes. They include the mainlobe level, backlobe, and all sidelobe levels. The highest peak will be always the main lobe level, followed in amplitude by the backlobe level or the highest sidelobe level, then the remaining peaks appear as secondary sidelobe levels. We always refer to the sidelobe level as the nearest peak to the mainlobe, while the backlobe level is the highest peak that falls behind the frontal scanning range of the array or that peak of the highest and farthest angular separation with the mainlobe direction, as shown in Figure 7a–g. The sidelobe levels are ranging from −12 to −13 dB, while the backlobe level can be as high as −8 dB at $\theta_{o}=0{}^{\circ}$ due to the symmetry of the cylindrical and concentric circular arrays in the structure. Therefore, it is recommended to reduce the size of the cylindrical array by minimizing their corresponding weights while maintaining the conical array and designing the concentric circular array at a minimum size, which may be one or two rings only.

### 3.3. Spatial Smoothing of CSC^4^ Power Pattern

The high sidelobe levels in the previous section can be reduced by adaptive tapered feeding. There are many tapered profiles that could be applied to the array currents [33,34,35,36,37], and in this paper, an adaptive oriented-peak cosine profile is proposed where the maximum coefficient values are always maintained at the nearest element to the mainlobe vector and the cosine is raised to a power that increases with $\theta_{o}$ to maintain lower sidelobe levels at higher elevation angles. The weighting vector orientation keeps the most affecting antenna elements at maximum projected area with the mainlobe direction, which effectively reduces the beamwidth.

The weights of the antenna array elements are determined according to their positions $\left(x_{m,n},\;y_{m,n},\;z_{m,n}\right)$, with respect to a central point $\left(x_{c},\;y_{c},\;z_{c}\right)$ resulting from the intersection of the mainlobe vector with the array surface, and this distance is given by:

(34) $$\rho_{m,n}=\sqrt{{\left(x_{c}-x_{m,n}\right)}^{2}+{\left(y_{c}-y_{m,n}\right)}^{2}+{\left(z_{c}-z_{m,n}\right)}^{2}}$$

So, the $mn^{th}$ element amplitude coefficient is proposed as follows:

(35) $$\alpha_{m,n}=\epsilon \left(\theta_{o}\right){\left(\cos\left(\frac{\pi \rho_{m,n}}{4\rho_{max}}\right)\right)}^{\left(3+\frac{4\theta_{o}}{\pi }\right)}$$

where $\rho_{max}$ is the maximum central distance between the central point on the array surface and its relatively farthest element in the array. $\epsilon \left(\theta_{o}\right)$ is a gain correction factor for direction dependency neutralization, which ensures constant maximum array gain is provided at any mainlobe direction and is needed to compensate for the loss in array gain due to both vector rotation and increase of tapering at higher values of $\theta_{o}$. $\epsilon \left(\theta_{o}\right)$ can be calculated from the following equation:

(36) $$\epsilon \left(\theta_{o}\right)=\frac{10^{\left(\frac{G_{p|t_{\max}}\;\left(\mathrm{dB}\right)-G_{p|t}\left(\theta_{o},\phi_{o}\right)\;\left(\mathrm{dB}\right)}{20}\right)}}{{\left(10^{\left(\frac{G_{p|t_{\max}}\;\left(\mathrm{dB}\right)-G_{p|t}\left(\theta_{o},\phi_{o}\right)\;\left(\mathrm{dB}\right)}{20}\right)}\right)}_{\max}}$$

where $G_{p|t_{\max}}\;\left(\mathrm{dB}\right)$ is the highest mainlobe gain and $G_{p|t}\left(\theta_{o},\emptyset_{o}\right)\;\left(\mathrm{dB}\right)$ is the array power gain in dB at any mainlobe direction calculated at $\epsilon \left(\theta_{o}\right)=1$, which is given by:

(37) $$\begin{array}{cc} G_{p|t}\left(\theta_{o},\phi_{o}\right)\;\left(\mathrm{dB}\right) & =G_{cyl|t}\left(\theta_{o},\phi_{o}\right)\;\left(\mathrm{dB}\right)+G_{con|t}\left(\theta_{o},\phi_{o}\right)\;\left(\mathrm{dB}\right)\\ & +G_{cc|t}\left(\theta_{o},\phi_{o}\right)\;\left(\mathrm{dB}\right) \end{array}$$

where

(38) $$G_{cyl|t}\left(\theta_{o},\phi_{o}\right)\;\left(\mathrm{dB}\right)=20\;\log\left(\overset{N_{cyl}}{\underset{n=1}{\sum }}\overset{M_{n}^{cyl}}{\underset{m=1}{\sum }}{\left(\cos\left(\frac{\pi \rho_{m,n}}{4\rho_{max}}\right)\right)}^{\left(3+\frac{4\theta_{o}}{\pi }\right)}\;\right)$$

(39) $$G_{con|t}\left(\theta_{o},\phi_{o}\right)\;\left(\mathrm{dB}\right)=20\;\log\left(\overset{N_{cyl}+N_{con}}{\underset{n=N_{cyl}+1}{\sum }}\overset{M_{n}^{con}}{\underset{m=1}{\sum }}{\left(\cos\left(\frac{\pi \rho_{m,n}}{4\rho_{max}}\right)\right)}^{\left(3+\frac{4\theta_{o}}{\pi }\right)}\right)$$

(40) $$G_{cc|t}\left(\theta_{o},\phi_{o}\right)\;\left(\mathrm{dB}\right)=20\;\log\left(\overset{N_{cyl}+N_{con}+N_{cc}}{\underset{n=N_{cyl}+N_{con}+1}{\sum }}\overset{M_{n}^{cc}}{\underset{m=1}{\sum }}{\left(\cos\left(\frac{\pi \rho_{m,n}}{4\rho_{max}}\right)\right)}^{\left(3+\frac{4\theta_{o}}{\pi }\right)}\right)$$

where $\theta_{o}$ in Equation (38) to Equation (40) is substituted in radians, while the azimuth angle $\phi_{o}$ can be dropped from these formulas due to the independency of the array power gain on it. 

For uniform feeding case, the array power gain $G_{p|uni}\left(\theta_{o},\phi_{o}\right)$ in dB is given by:

(41) $$G_{p|uni}\left(\theta_{o},\phi_{o}\right)\;\left(\mathrm{dB}\right)=20\;\log\left(\eta_{T}\right)$$

The normalized power gain is used for investigating the relative radiation levels such as sidelobes and backlobe with respect to the mainlobe level, however, for real-world communication scenarios, the absolute power gain of the array is meaningful and should be utilized to achieve the required performance level. 

To perform weighting vector orientation, the central point $\left(x_{c},\;y_{c},\;z_{c}\right)$ is chosen as the coordinates of the nearest element in the array to the mainlobe direction using the following sequence of equations:

(42) $$\theta_{c}=\theta_{n},\;at\;{\left|\theta_{n}-\theta_{o}\right|}_{min}$$

(43) $$\phi_{c}=\phi_{m,n},\;at\;{\left|\phi_{m,n}-\phi_{o}\right|}_{min}$$

with n determined at $\theta =\theta_{c}$,

(44) $$R_{c}=R_{n},\;at\;{\left|\theta_{n}-\theta_{o}\right|}_{min}$$

Finally, the weighting vector of the array can be written as follows:

(45) $$W\left(\theta_{o},\phi_{o}\right)=\alpha_{m,n}A_{S}\left(\theta_{o},\phi_{o}\right)$$

**Figure 8:**
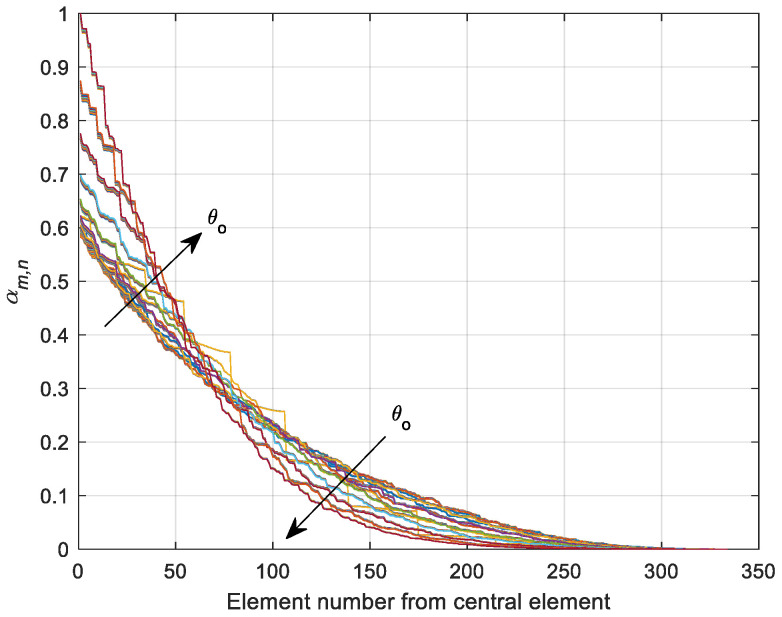
Variation of the array amplitude coefficients with the mainlobe direction. The arrows refer to the curves at higher values of $\theta_{o}$.

Figure 8 displays the tapered variation of $\alpha_{m,n}$ with the elements at different elevation angles $\theta_{o}$ where the arrow in this figure is directed toward the higher values of $\theta_{o}$. The array becomes more tapered if $\theta_{o}$ increases where the array elements become more self-blocked by its interior body structure. For this reason, the contribution of blocked elements should be minimized with higher tapering exponent that should increase with $\theta_{o}$. On the other hand, the correction done by $\epsilon \left(\theta_{o}\right)$ should lead to coefficients’ amplitudes less than or equal to 1 where no amplification is recommended during weighting.

**Figure 9:**
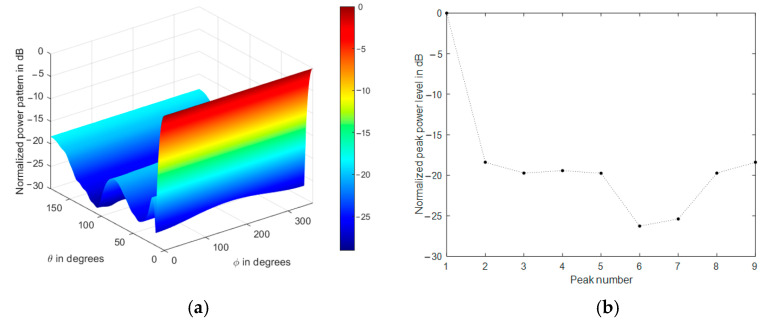

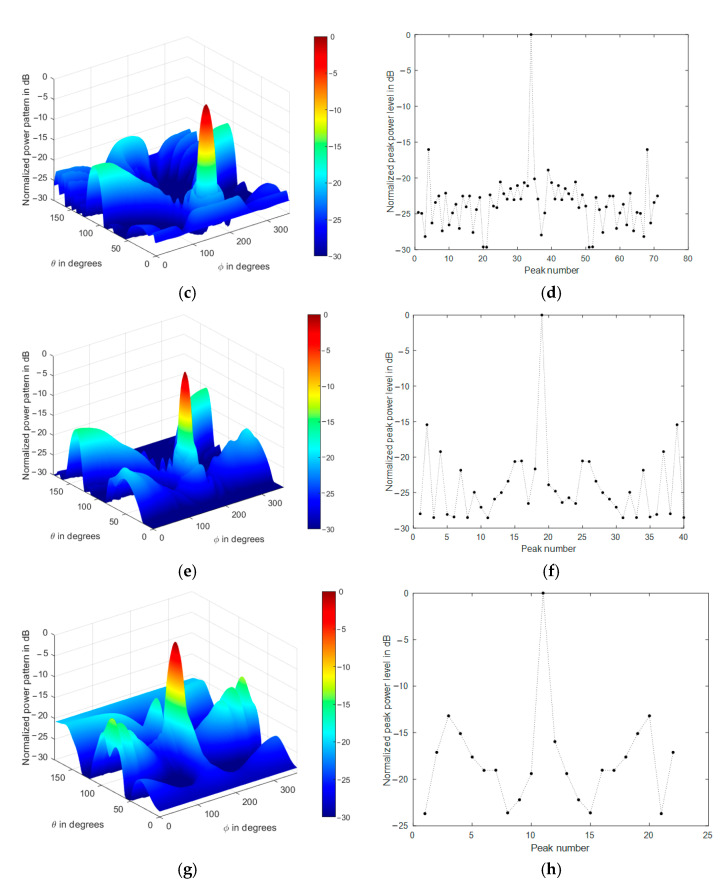
Normalized power pattern of spatially filtered CSC^4^ (**a**,**c**,**e**,**g**) and the corresponding radiation peaks (**b**,**d**,**f**,**h**) at four different mainlobe directions (0°,180°), (30°,180°), (60°,180°), (90°,180°), respectively.

**Figure 10:**
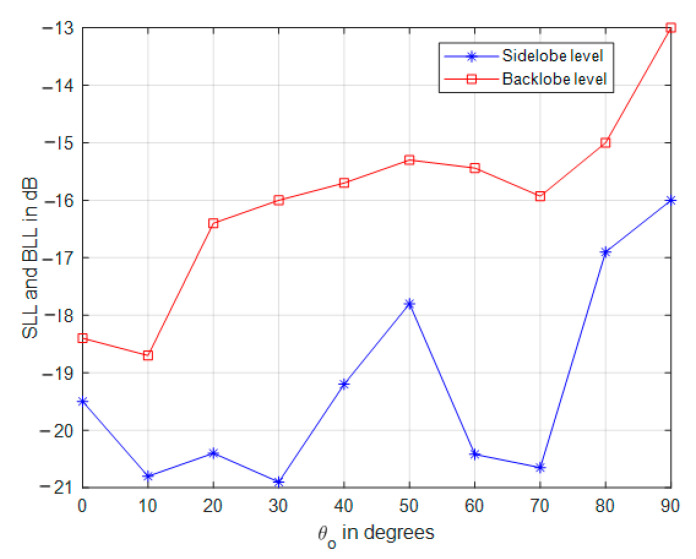
Sidelobe and backlobe variation with the mainlobe direction.

The impact of tapered amplitude weighting in Equation (42) results in a phased array with lower sidelobe levels, as shown in Figure 9 for the same array parameters in the previous section. The number of peaks in the power pattern is also reduced due to spatial smoothing; in addition, the backward radiation is reduced, which is very important in many applications which are affected by interference. The variation of sidelobe and backlobe levels with the mainlobe direction are shown in Figure 10, which depicts the low backlobe levels over a wide range of scanning mainlobe directions with reduced sidelobe levels. 

### 3.4. 3 dB Beamwidth Performance of CSC^4^ Array

**Figure 11:**
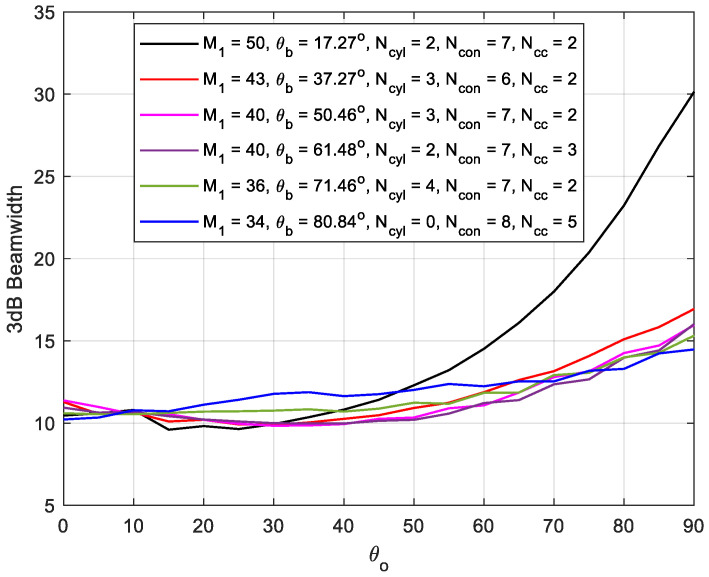
Half-power beamwidth variation with mainlobe direction at different CSC^4^ array designs with fixed array size of 334 elements.

In this section, the array 3 dB beamwidth is investigated at variable mainlobe direction and conical base angle. The total number of the array elements is kept constant and the whole number of the array elements is utilized in different designs to investigate the impact of base angle on the beamwidth performance. The results are displayed in Figure 11, where, generally, increasing $\theta_{o}$ increases beamwidth, especially at lower values of $\theta_{b}$. The curves in this figure are not smoothly varying due to the weighting profile used in Equation (35). An important feature of CSC^4^ array is that the beamwidth is almost constant for mainlobe directions ranging from 0° to 70° with only ±1° variation, which is due to the almost constant projected area of the array along with the weighting vector orientation in Equation (35). At lower conical array base angles, the beamwidth increases rapidly due to the flatness of the array surface, which is affected by the projection at higher values of $\theta_{o}$ along with the interaction of the mainlobe, with the secondary backlobe leading to more wider beams. 

## 4. Frontal Mainlobe-Oriented Partial CSC^4^ Array Beamforming

**Figure 12:**
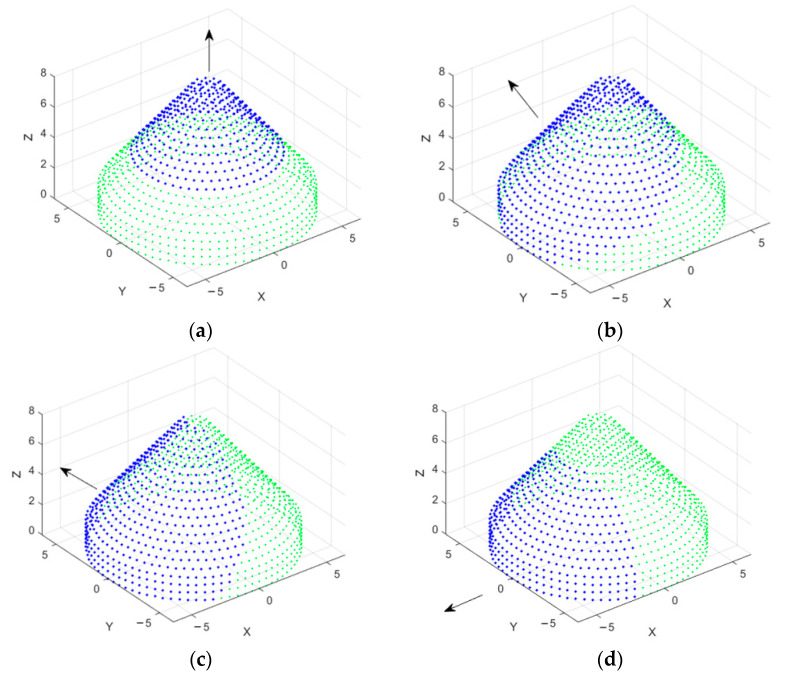
Frontal mainlobe-oriented partial CSC4 concept. The green dots represent the whole CSC4 array; (**a**) partial array at $\theta_{o}=0{}^{\circ}$, (**b**) partial array at $\theta_{o}=30{}^{\circ}$, (**c**) partial array at $\theta_{o}=60{}^{\circ}$, (**d**) partial array at $\theta_{o}=90{}^{\circ}$.

At higher frequencies, the antenna elements are miniaturized, so when fixing conformal antenna arrays on large body structures such as space shuttles or rocket boasters, the size of the array will be very large, and the beamforming operation becomes very consumptive in processing. On the other hand, the overall array spatial power pattern also depends on both individual element power pattern and array factor. According to the type of antenna used, some elements possess low radiation pattern toward the backside of the element, which means that the element is effective only in the frontal directed beams, instead of any other directions. In addition, the body in which the array is fixed will provide blocking effects on the array patterns, and the most effective array response will be in the half-spherical region centered around the mainlobe direction. Therefore, in this section, the frontal partial CSC^4^ antenna array is proposed and investigated, where a partial frontal array centered around the mainlobe direction is selected from the whole CSC^4^ array structure, as shown in Figure 12. The other backward antenna elements in the CSC^4^ are muted, as they will be blocked by the conformed body. The size of the partial array can be controlled by a threshold distance measured from the center of the partial array to the antenna elements that form the CSC^4^ array according to the required radiation pattern properties, such as beamwidth and mainlobe gain.

The antenna elements in this subarray can be weighted using Equation (35), where the maximum amplitude is kept at the central element of the partial array which is nearest to the direction of the mainlobe, while the lowest amplitude weight is assigned to the elements at the subarray boundary. In addition, the partial array can be modified so that its center is always located on the mainlobe direction to provide maximum frontal effective array area, which results in improved array radiation pattern and smaller beamwidth values. However, the flatted surface of this array may result in higher backlobe radiation levels. As a case study, the whole CSC^4^ array is designed with a larger number of elements, but the selected partial array is always kept at a fixed size of 334 elements. 

The threshold distance, which defines the subarray boundary, is adapted to maintain the total number of elements constant at any mainlobe direction. So, the threshold distance, $\gamma_{p}$, has the following limits:

(46) $$0<\gamma_{p}\leq \rho_{max}$$

**Figure 13:**
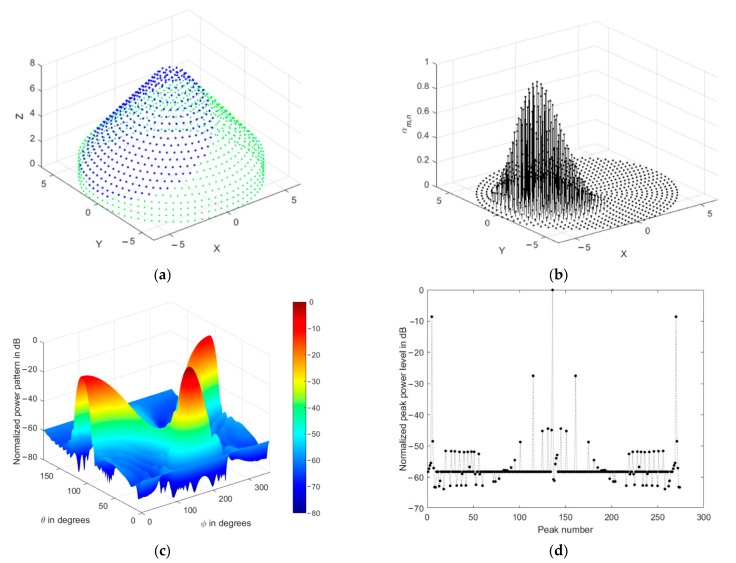
Frontal mainlobe-oriented partial CSC^4^: (**a**) partial array active antenna elements (blue dots), (**b**) amplitude coefficients, (**c**) normalized power pattern (with −50 dB floor), and (**d**) radiation peak levels.

To utilize the whole CSC^4^ array, we should set the threshold to $\gamma_{p}=\rho_{max}$. Increasing the threshold value will utilize a larger number of elements, which is required to obtain smaller beamwidths, especially at mainlobe directions near $\theta_{o}=90{}^{\circ}$. Figure 13 displays the mainlobe-oriented partial CSC^4^ array extracted for a mainlobe directed toward (30°,180°) with $M_{1}^{cyl}=80$, $N_{cyl}=3$, $N_{con}=18$, $N_{cc}=2$, $\eta_{T}=334$, and $\gamma_{p}=2.5\lambda$. The green dots in this figure refer to the unused antenna elements, while the blue ones correspond to the active subarray. 

**Figure 14:**
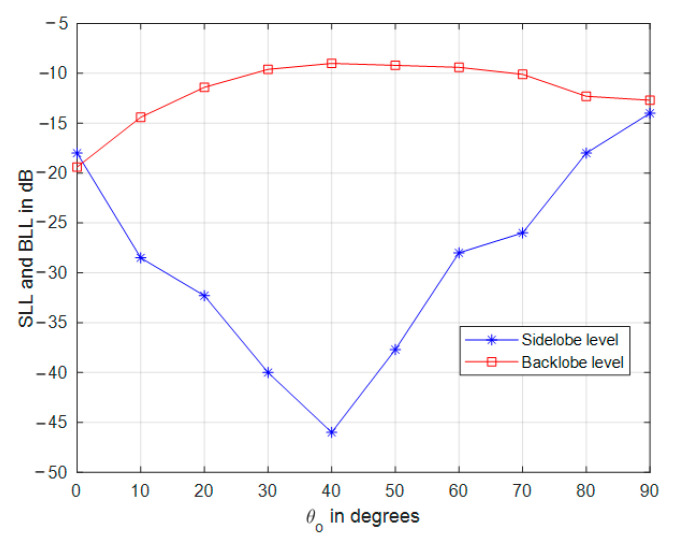
Sidelobe and backlobe variation with the mainlobe direction.

Figure 13 displays the amplitude coefficients for the whole CSC^4^ array with their projected locations on the XY-plane, while Figure 14 displays the corresponding normalized power pattern and radiation peaks, respectively.

An interesting feature of the frontal mainlobe-oriented partial CSC^4^ array is that there is almost one mainlobe and some shoulders in the radiation pattern at deep levels, and one backlobe with a reduced level. The radiation shoulders may appear in higher levels than the first sidelobe level, so we may choose the shoulder level instead of the first sidelobe level in this case. The mainlobe orientation may be extended to values of $\theta_{o}>90{}^{\circ}$ using the same frontal partial array at $\theta_{o}=90{}^{\circ}$ and it still works; however, it is better to use the designed array in the range of $-90{}^{\circ}\leq \theta_{o}\leq 90{}^{\circ}$. The performance of the sidelobe and backlobe radiation levels with the mainlobe direction at any azimuthal angle is shown in Figure 14 in the frontal directions (i.e., at $-90{}^{\circ}\leq \theta_{o}\leq 90{}^{\circ}$) while keeping the threshold distance at 2.5λ.

The power pattern of the partial CSC^4^ array has a very efficient frontal radiation pattern, especially for slanted angles where the sidelobe levels fall very deep with reduced backlobe radiation levels of about −10 dB. However, this array design suffers from slightly higher sidelobe levels at angles near to $\theta_{o}=0{}^{\circ}$ and 90°. For a mainlobe directed toward $\theta_{o}=0{}^{\circ}$, the array is composed completely from circular and conical arrays, while at any other look angle, the array is formed partially from the three stacked subarrays, especially when $\theta_{o}=90{}^{\circ}$ and $\gamma_{p}>2.5\lambda$. At mainlobe directions other than $\theta_{o}=0{}^{\circ}$ or 90°, the array provides very low sidelobe levels, which can be down to −45 dB. This conformal array beamforming may be applied in cases where the individual antenna elements possess a kind of directional pattern and low backward radiation, so the increased secondary backlobe radiation of the array does not affect the array overall response and radiated power. Therefore, this partial CSC^4^ array can provide more flexible design solutions if massive CSC^4^ array is available, where the extracted subarray can be adaptively selected according to the mainlobe direction and sidelobe levels required for a specific application. 

Table 4 summarizes the performances of different array structures and the proposed CSC^4^ according to the simulation results obtained in the paper. For comparison purposes, the total number of elements in each array is kept constant and all arrays are fed by the same feeding profile in Equation (35). The performance of the concentric circular array is superior only regarding sidelobe level, especially at $\theta_{o}=0{}^{\circ}$, however it has the highest backlobe level which is the same level as the mainlobe (0 dB). The weighting profile orientation with the mainlobe direction in the partial CSC^4^ has a very positive effect on the performance, which is the almost constant beamwidth over very wide scanning angles, as shown in Table 1, compared to any other array structure. Also, this array provides very acceptable sidelobe and backlobe levels compared to the conical array structure, especially for midrange angular orientation of the mainlobe.

The antenna elements in the CSC^4^ are assumed to be isotropic radiators, however, other practical directional antennas can be utilized where it is expected that the overall radiation pattern will be further improved, especially the backlobe and sidelobe levels. Additionally, mounting the antenna elements to the conformed surface always makes the broadside direction of each element perpendicular to that surface which achieves the wide scanning angular range capability. 

**Figure 15:**
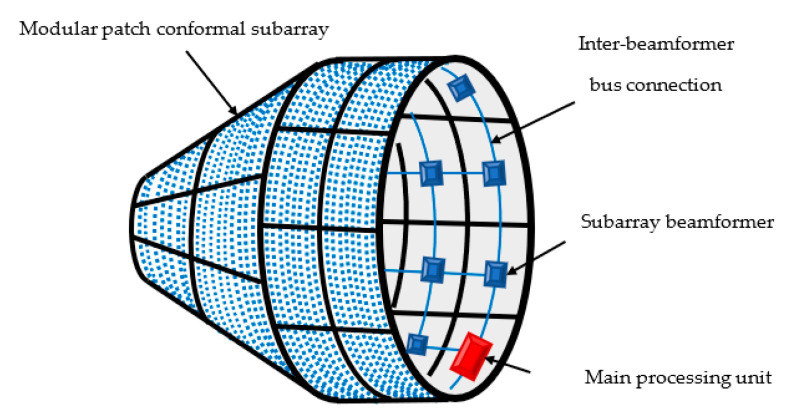
Hypothetical configuration of large conformal CSC^4^ array and beamforming network.

The beamforming system includes a controlled set of phase shifters and attenuators (which are used to implement the weight values in Equation (35)) and should be properly connected to the external antenna elements. These components are controlled by a beamforming processor according to the mainlobe direction, gain, and beamwidth. The large-size antenna array mounted on large, conformed bodies may be installed in a modular patched form of integrated subarrays. This modular structure eases the fabrication and fixing operation of large conformal arrays, as shown in Figure 15. The beamforming network can be connected to the external antenna elements of each subarray from the interior of the conformed body through holes by cables, as done in most space vehicles and airplanes. In addition, the beamforming system can be split into beamforming subsystems in the case of very large structures, such as space vehicles, where it is almost practically impossible to connect all the array elements to one beamforming network unit. In this case, synchronized parallel processing should be done in the beamforming subsystems where the overall output is obtained from combining the outputs of each local beamformer by a main processing unit.

## 5. Impact of Nonisotropic Antenna Elements on the Radiation Pattern

In the previous sections, we adopted isotropic antenna elements in the CSC^4^ array to investigate the performance of the proposed beamforming technique and its impact on the sidelobe and backlobe levels as well as the scanning capabilities and beamwidth stability. However, other directional antenna elements can be used, as is discussed in this section. Some antenna designs utilize metallic ground planes (perfect electrical conductors (PEC)) or are mounted on the metallic structure of the conformed body, which affects the 3D radiation pattern of the whole array. The radiation pattern in this case is expected to have very low backlobe radiation, which is further reduced by the proposed technique which increases the security and signal quality in various communications scenarios, such as space, radar, and remote sensing applications. Additionally, the practical antenna elements will be subjected to mutual coupling, which mainly arises from the laterally radiated fields towards the neighboring elements, especially when the required mainlobe direction is near or parallel to the array surface. However, the frontal mainlobe-oriented beamforming technique proposed in the last section may reduce the mutual coupling by adopting the mainlobes of the directional antenna elements in the desired scanning range, where the partial array is formed by selecting the antenna elements that are mostly facing the desired mainlobe direction. Additionally, the rounded structure of the conformed UAV or spacecraft body provides symmetrical antenna distribution around the body axis, and the selected partial array will therefore generate symmetrical scanning beams, and no phasing is required for the antenna elements to generate beams with inclined angles to their surfaces. Indeed, this concept is utilizing the maximum effective area from antenna elements. If this technique is jointly applied with antenna elements that are properly interisolated by absorptive walls such as the metamaterial isolation walls [38,39], then the radiated fields towards neighboring elements is minimized, and the mutual coupling effect is minimized as well. Furthermore, the rounded conformal surface results in rapid decaying of the mutual coupling, as shown in [40]. 

### 5.1. Performance of CSC^4^ Array Using Crossed Dipole Antennas with Ground Plane

As there are many antenna structures that can be used to construct the array, we may test the radiation pattern of the frontal mainlobe-oriented CSC^4^ array using crossed dipole antenna (turnstile antenna) with metallic ground plane. The simulation parameters are listed in Table 5, using the same array configuration as in Section 4 for comparison purposes. These parameters are used along with the array feeding function in Equation (35) to form a sample of mainlobe that is directed towards (30°,180°) as input information to the simulation package, which utilizes the phased antenna arrays toolbox in MATLAB or Ansys HFSS and helps test conformal arrays of various antenna element types including mutual coupling effects.

**Figure 16:**
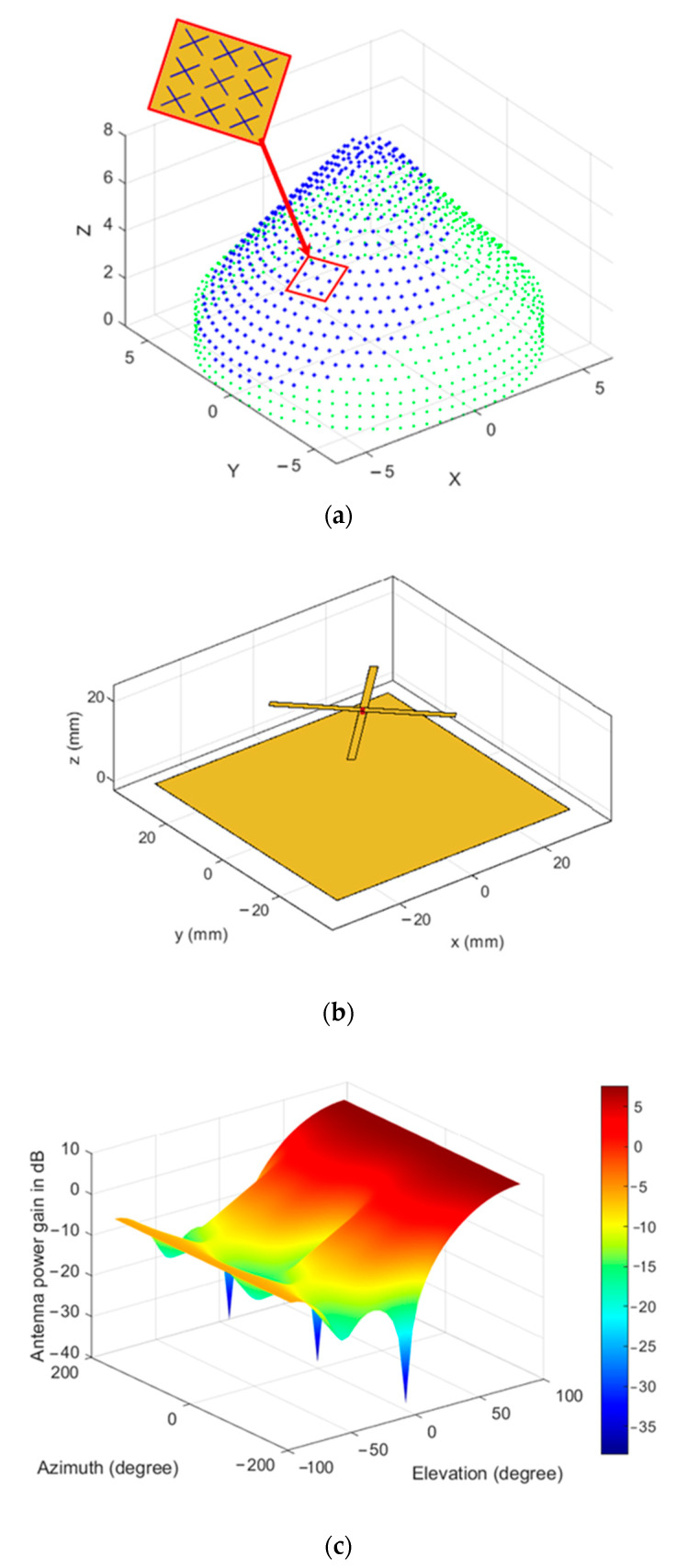
Crossed dipole antenna (turnstile antenna) with ground metallic plane: (**a**) complete CSC^4^ array with active elements in blue colored dots and distances normalized to wavelength, (**b**) single crossed dipole element structure at frequency of 3.5 GHz, and (**c**) is the single antenna element radiation pattern.

As shown in Figure 16a, the partial mainlobe-oriented partial CSC^4^ is formed by mounting crossed dipole antennas on the conformed surface which may be made from metal or other materials, so we consider that each crossed dipole antenna element has its own finite ground plane which is made from PEC material. Additionally, if the conformed structure is made from PEC, then we can consider that the crossed dipole element is mounted on a very large ground plane in this case. The two cases of finite and very large ground plane are both tested. Figure 16b displays the geometric structure of the crossed dipole element which is formed by two half-wavelength dipoles that are right angled at the center and fixed over a metallic ground plane at quarter-wavelength separation distance. The proposed ground plane has an area of 0.75λ×0.75λ as designed in [41], and almost provides an isotropic radiation pattern in the upper hemisphere where the elevation angle is ranging from 0° to 90°, while minimizing the backlobe radiation in the other hemisphere, as depicted in the radiation pattern shown in Figure 16c. The front-to-back ratio of this antenna element is 13.3 dB and has a maximum gain of 7.6 dB and is in the boresight direction that is perpendicular to the ground plane and passing through the antenna center, while the backlobe level is approximately at −5.7 dB with a 3 dB beamwidth of approximately 100°. Increasing the ground plane area will reduce the backlobe radiation significantly [41], therefore, for large UAVs and space vehicles, the metallic body structure around which the antenna elements are fixed provides a very large ground plane, and therefore produces negligible backlobe radiation. 

**Figure 17:**
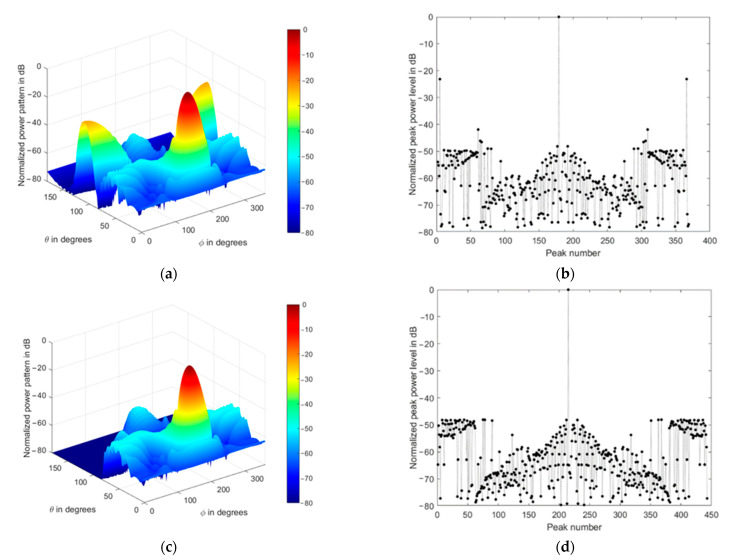
Frontal mainlobe-oriented partial CSC^4^ with practical elements (**a**) radiation pattern of crossed diploe antenna array with finite metallic ground plane, (**b**) radiation peaks for the element in (**a**), (**c**) radiation pattern of crossed diploe antenna array with very large metallic ground plane, (**d**) radiation peaks for the element in (**c**). All radiation patterns are clipped at −80 dB for a clearer view and comparison purposes.

In Figure 17, the array is tested using nonisotropic antenna structures to generate a mainlobe in the same direction of that generated previously in Figure 13, which is (30°,180°), and with the same array configuration, parameters, and feeding profile calculated from Equation (35) for the purpose of comparison. Figure 17a,b depict the impact of using the crossed dipole antenna elements as designed in Figure 16, where the backlobe radiation is further reduced by 13.3 dB, plus a 10 dB reduction resulted from the proposed beamforming technique, so the backlobe is now at approximately −23 dB relative to the mainlobe level. If we consider the metallic body structure as a ground plane for all antenna elements, then this large structure will further reduce the backlobe radiation, as shown in Figure 17c,d, where the sidelobes and backlobes are damped to very low levels of −48 dB, relative to the mainlobe level. The mutual coupling effect appears as some increase in the mainlobe width but at low radiation levels near −40 dB, however, the 3 dB beamwidth is almost not changed with respect to the isotropic radiator case shown previously in Figure 13c due to the almost isotropic radiation pattern of the crossed dipoles around their broadside direction. 

### 5.2. Comparsion with Relevant Conformal Array Designs

As shown previously in Figure 14, the proposed conformal array in this paper provides its best performance at mainlobe directions near its broadside, which can be considered as the same as the cone base angle; however, the concept of orienting the array feeding amplitude so that the maximum amplitude weighting is assigned to the elements that are near to or located on the mainlobe vector minimizes the beamwidth variation and hence provides efficient beam scanning. To clearly demonstrate the contribution of the current work, we compare the performance of the proposed stacked array with the most relevant recent work presented in [6,20], where they almost aim at the same objectives and array structure. While there many other proposed airborne antenna systems, they do not provide common objectives that may be included in the comparison which mainly provides fixed power patterns in a broadside or endfire direction without beam steering capabilities or optimization. In [6], the array is formed by conical surface with slots which are utilized partially, and the feeding is optimized using genetic algorithm, while in [20] the array consists of conical and cylindrical arrays which are implemented partially using microstrip antenna elements which are fed with amplitude tapering profile. The three structures are compared in Table 6, including their structures, capabilities, and key performance parameters, such as the mainlobe gain, sidelobe level, beamwidth, and scanning range. The comparison shows that the proposed stacked array provides more stable and almost invariant beamwidth in the scanning range, compared to the other structures. Additionally, the proposed array provides a much lower sidelobe level at the broadside direction with slightly higher mainlobe gain that is 2 dB more than the technique in [6] and 6 dB higher than provided in [20]. In addition, it is found that the proposed array provides −40 dB with the same number of elements and base radius of the other two designs. On the other hand, at the same number of elements and base radius, the proposed stacked array provides 12° lower beamwidth than in [20], but is higher than [6] by 4°. Therefore, we can conclude that the proposed stacked array has efficient scanning performance with almost constant beamwidth due to the proposed weighting function in Equation (35) which also reduces the sidelobe levels. As expected, the inclusion of concentric circular array at the front of the proposed array improves the mainlobe gain.

## 6. Practical Challenges Discussion

The flexibility and high gain at low sidelobe and backlobe levels of the proposed CSC^4^ can be achieved practically for UAV communication systems when inserting the antenna pattern with the array factor in Equation (25). However, fixing antenna arrays on the outer surface of the vehicle body for UAV, and especially for space vehicles, is faced by some challenges. For example, different values from the cone base angle, $\theta_{b}$, listed in Table 1 may be used for body structures with different cone base angles resulting in minor changes of the interelement spacing which will not seriously impact the overall radiation pattern. On the other hand, for a space vehicle, the different atmospheric environments where it is passing through are very challenging for conformal arrays. Therefore, special materials should be used in manufacturing and protecting the conformal array that should tolerate the huge variance in temperature, air pressure, and aerodynamics during launch stages. Additionally, the exposed surface of the conformal array structure should not affect the aerodynamic performance of the vehicle. However, the in-orbit performance will be much better than during launch phases where there are no atmospheric and aerodynamic problems. 

## 7. Conclusions

As the conformal arrays are very important in many applications, especially for airborne, UAVs, and space vehicles, this paper proposes an efficient design and beamforming technique for these systems using a stacked or modular structure concept where cylindrical, conical, and concentric circular arrays are used. The array structure is built starting with a base of cylindrical array followed by conical array and ended with concentric circular array where the whole structure is conformal to the tapered vehicle front or back. The array is modeled geometrically, and the corresponding steering vector is deduced. The radiation pattern performance is also described, starting with uniform feeding, and is improved by a proposed feeding profile which is adaptively determined by the mainlobe direction. This weighting profile reduces the sidelobe and backlobe levels and provides wider scanning angular range by adjusting the maximum feeding amplitude coefficient so that it is always located at the central element that is oriented with the mainlobe direction. In addition, for large array structures, a frontal mainlobe-oriented partial CSC^4^ array beamforming is proposed to effectively select a subarray that has the maximum effective area to provide lower sidelobe levels with reduced backlobe radiation and almost constant beamwidth over a wide angular range. Simulation results show that almost constant backlobe levels at −10 dB at any direction in the frontal range (i.e., $-90\leq \theta_{o}\leq 90{}^{\circ}$) can be achieved with sidelobe levels that can be reduced to −45 dB at the center of the scanning range. In the future work, this array structure will be further manipulated and practically implemented to be applied in civil aviation systems to improve the aerial-to-ground (A2G) and aerial-to-air (A2A) aeronautical telecommunication networks. 

## Figures and Tables

**Table 1:** Inter-ring number of elements variation and the corresponding base angles for uniform conical array design.

l	1	2	3	4	5	6
$\theta_{b}$	80.84°	71.44°	61.48°	50.46°	37.27°	17.27°
$\Delta M=M_{n+1}^{con}-M_{n}^{con}$	1	2	3	4	5	6

**Table 2:** Individual array design parameters for comparison.

Array Type	Design Parameters
Conical	$\theta_{b}=50.16{}^{\circ}$, $M_{1}^{con}=27$, $N_{con}=7$, $\eta_{T}^{con}=105$
Cylindrical	$\theta_{b}=90{}^{\circ}$, $M_{1}^{cyl}=21$, $N_{cyl}=5$, $\eta_{T}^{cyl}=105$
Concentric Circular	$\theta_{b}=0{}^{\circ},$$M_{1}^{cc}=33$, $N_{cc}=6$, $\eta_{T}^{cc}=105$

**Table 3:** CSC^4^ arbitrary array parameters for power pattern testing.

Array Parameter Values	$M_{1}^{cyl}$	$N_{cyl}$	$N_{con}$	$N_{cc}$	$\eta_{T}$	$\theta_{b}$
	40	3	7	2	334	50.46°
$\left(\theta_{o},\phi_{o}\right)$	(0°,180°), (30°,180°), (60°,180°), (90°,180°)					

**Table 4:** Summary of array performance for different array structures and the proposed CSC^4^ and partial CSC^4^.

Array Structure	Sidelobe Level (dB)			Backlobe Level (dB)			Beamwidth (Degrees)		
$\theta_{o}$	0°	45°	90°	0°	45°	90°	0°	45°	90°
Conical array	−24	−32	−32	−18	−17	−11	13°	15°	18°
Cylindrical array	−8	−9	−21	0	−14	−9	7°	8°	23°
Concentric circular array	−37	−40	−40	0	0	−15	11°	14°	48°
CSC^4^	−20	−19	−16	−18	−16	−13	11°	10°	14°
Partial CSC^4^	−19	−42	−14	−20	−10	−13	10°	10°	14°

**Table 5:** Simulation parameters for frontal-oriented mainlobe partial CSC^4^ using crossed dipole as an antenna element.

Simulation Parameter	Value
Operating frequency	3.5 GHz
Wavelength, *λ*	85.7 mm
Antenna elements interseparation distance	0.5λ
Number of elements of the base cylindrical array	80
Conical array base angle	50.46°
Number of cylindrical arrays	3
Number of conical arrays	18
Number of concentric circular array	2
Partial CSC^4^ array radius, $\gamma_{p}$	2.5λ
Number of active elements in the partial CSC^4^ array	334
Dipole length	40.81 mm
Dipole width	1.71 mm
Dipole feeding location	Center
Ground plane material	PEC
Ground plane dimensions	0.75λ×0.75λ
Separation between the crossed dipole and ground plane	0.25λ
Dielectric material	Air
Array base diameter	1091 mm
Array height	857 mm

**Table 6:** Simulation parameters for frontal-oriented mainlobe partial CSC^4^ using crossed dipole as an antenna element.

Comparison Item	This Work	Ref. [6]	Ref. [20]
General array structure	Concentric circular, conical, and cylindrical	Conical only	Conical and cylindrical
Mainlobe-oriented partial array formation	Yes	Not examined	No
Maximum amplitude always at the nearest element to the mainlobe direction	Yes	No	No
Elevation scanning range at almost constant beamwidth	−70°≤θ≤70°	−40°≤θ≤120°	0°≤θ≤30° at 30°≤ϕ≤150°, 30°≤θ≤180° at 60°≤ϕ≤150°
Beamwidth variation over scanning range	~1°	~4°	~15°
Azimuth scanning range	−180°≤ϕ≤180°	−60°≤ϕ≤60°	30°≤ϕ≤150°
Beamwidth at broadside direction	Determined at the same total number of elements and base radius of the compared array	14° Proposed array beamwidth = 18° at 117 elements and with cylindrical base radius of 3λ	30° Proposed array beamwidth = 18° at 88 elements and with cylindrical base radius of 6.9λ
Highest sidelobe level relative to a mainlobe formed at the array broadside direction	−40 dB	−30 dB	−15.5 dB
Mainlobe gain in dB	26.04 dB	24 dB	20 dB
Antenna elements feeding method	Adaptive cosine exponent tapered amplitude feeding	Genetic algorithm	Constant tapered amplitude feeding
Element design	Crossed dipole antenna with PEC ground plane	Slots on conical surface	Microstrip antenna

## Data Availability

Data sharing not applicable.
